# Exploring the relationship between proactive inhibition and restrictive eating behaviours in severe and enduring anorexia nervosa (SE-AN)

**DOI:** 10.1186/s40337-024-01165-y

**Published:** 2025-01-03

**Authors:** Savani Bartholdy, Bethan Dalton, Samantha J. Rennalls, Maria Kekic, Jessica McClelland, Iain C. Campbell, Owen G. O’Daly, Ulrike Schmidt

**Affiliations:** 1 Centre for Research in Eating and Weight Disorders, Department of Psychological Medicine, Institute of Psychiatry, Psychology & Neuroscience, King’s College London, London, UK; 2 Centre for Neuroimaging Sciences, Department of Neuroimaging, Institute of Psychiatry, Psychology & Neuroscience, King’s College London, London, UK; 3 South London and Maudsley NHS Foundation Trust, London, UK

**Keywords:** Anorexia nervosa, Proactive inhibition, Inhibitory control, Restrictive eating, Intolerance of uncertainty

## Abstract

**Background:**

There is a need for improved understanding of why 20–30% of individuals with anorexia nervosa (AN) develop a severe and enduring form of illness (SE-AN). Previously, we reported differences in proactive inhibition (a pre-emptive slowing of responses) in individuals with AN compared to healthy controls (after controlling for intolerance of uncertainty). The present study is a preliminary exploration of proactive inhibition in which we compared women with SE-AN with healthy comparison (HC) women and explored its association with restrictive/avoidant eating behaviours.

**Methods:**

Thirty-four women with SE-AN (defined by >3 years of illness and a previous unsuccessful course of eating disorder treatment) and 30 HCs completed (a) a cued reaction time task, to assess proactive inhibition, and (b) questionnaires assessing restrictive/avoidant eating behaviours and intolerance of uncertainty.

**Results:**

Both SE-AN and HC participants showed slower reaction times under conditions of uncertainty, indicating proactive inhibition in both groups. There was a main effect of group, with SE-AN participants showing significantly slower reaction times compared to HC. There was no interaction between group and condition, suggesting that individuals with SE-AN did not differ in proactive inhibition compared to HCs. However, post-hoc analysis between-group tests for each trial type revealed that group differences were only present under conditions of uncertainty. Proactive inhibition was not significantly associated with self-reported restrictive/avoidant eating behaviours, including when taking intolerance of uncertainty into consideration.

**Conclusions:**

It is unlikely that proactive inhibition contributes to avoidant and restrictive eating behaviours seen in SE-AN. Our findings suggest that the SE-AN group are relatively more cautious when responding under conditions of uncertainty. Longitudinal studies and between-group comparisons of individuals across different stages of illness will be required to elucidate the way in which proactive inhibition is specifically implicated in SE-AN, rather than in AN more generally.

**Supplementary Information:**

The online version contains supplementary material available at 10.1186/s40337-024-01165-y.

## Abstract (plain language summary)

Proactive inhibition refers to the pre-emptive slowing of responses. We assessed whether 34 women with severe and enduring anorexia nervosa (defined by greater than 3 years of illness and a previous unsuccessful course of treatment) differed from 30 healthy comparison women in proactive inhibition. We also explored if proactive inhibition was related to restrictive eating behaviours. All participants completed a cued reaction time task to assess proactive inhibition and questionnaires assessing restrictive/avoidant eating behaviours and intolerance of uncertainty. All participants showed proactive inhibition, i.e., slower reaction times under conditions of uncertainty. Participants with severe and enduring anorexia nervosa showed significantly slower reaction times compared to healthy comparison women. Proactive inhibition was not significantly associated with self-reported restrictive/avoidant eating behaviours. Our findings suggest that compared to the healthy comparison group of women, the severe and enduring anorexia nervosa group are more cautious when responding, specifically in the context of uncertainty. It is unlikely that proactive inhibition contributes to avoidant and restrictive eating behaviours in severe and enduring anorexia nervosa.

**Supplementary Information:**

The online version contains supplementary material available at 10.1186/s40337-024-01165-y.

## Introduction

Anorexia nervosa (AN) is characterised by restricted food intake and inappropriate weight-loss behaviours (e.g., excessive exercise), accompanied by a fear of weight gain and body dissatisfaction [1]. Evidence-based treatments are moderately effective [2]. However, ~ 20–30% of patients develop a severe, enduring form of illness (SE-AN) [3, 4], for reasons that are unclear. For this group, there are limited treatment options [5] and new interventions are needed [6] Neurocognitive studies may provide insight into mechanisms underlying the more longstanding form of the illness and inform how treatments can be tailored or developed for this group [6, 7].

Inhibitory control, which refers to the ability to appropriately withhold a response, has been implicated in AN. Indeed, data suggest that people with AN display excessive inhibitory control over their behaviour [8] and it is possible that inhibitory control may be one mechanism through which persistent food restriction is achieved. However, inhibitory control is a complex behaviour, and it would be beneficial to explore ways in which aspects of it may be involved in AN (see [9] for theoretical review). We have proposed [9] that a proactive form of behavioural inhibitory control referred to as proactive inhibition (i.e., a *pre-emptive* withholding or slowing of responses under uncertainty) may be relevant to AN. Behaviourally, this relates more to the preparatory or strategic slowing of responses when the outcome is unknown or uncertain [9, 10]. For example, this may reflect the extent to which warning cues can benefit response times (‘warning benefit’ e.g., an amber traffic light indicating the upcoming need to stop at an intersection), or the extent to which responses slow when the need for a response is uncertain (‘preparation cost’, e.g., driving more slowly when passing schools in case a child runs into the street).

Research on proactive inhibition in AN is in its infancy. We have published findings of an initial study comparing proactive inhibition across individuals with AN, bulimia nervosa (BN), binge-eating disorder (BED) and healthy controls [11]. We found mixed support for the involvement of proactive inhibition as a potential underlying mechanism in AN: all groups demonstrated proactive inhibition (i.e., slower responses under conditions of uncertainty), however a main effect of group only emerged after covarying for intolerance of uncertainty. This finding appeared to be driven predominantly by slower response times in the AN group only during trials involving uncertainty. To our knowledge, only one other study has explored proactive inhibition in individuals with AN: Westwater et al. [12] found that, relative to a healthy comparison group, individuals with AN binge-eating/purging type did not differ in the behavioural assessment of proactive inhibition, but demonstrated greater activity in the right inferior frontal gyrus during proactive inhibition.

As proactive inhibition involves adjusting one’s response speed and preparedness to respond, it is likely to be related to decision making in the context of uncertainty, i.e., how one adjusts responding when the need for a response is less certain. For example, individuals may prioritise accuracy and delay responses until further evidence has been gathered. People with AN are reported to be less tolerant of uncertainty than healthy individuals [13, 14] and therefore, may exercise proactive inhibition more strongly in the context of uncertainty. Research has shown that people with AN engage in dietary restriction and behavioural avoidance in efforts to reduce the anxiety and negative feelings caused by uncertainty [15–17] and intolerance of uncertainty has been associated with greater AN symptom severity [18–20]. It is possible therefore that in the context of uncertainty, proactive inhibition may promote the use of more restrictive and/or avoidant behaviours. Accordingly, in the present study, we have explored whether proactive inhibition relates to avoidant/restrictive eating behaviours in SE-AN.

As few studies have examined neurocognitive factors related to SE-AN, we sought to replicate our previous study [11] in an independent and well-characterised sample of individuals with SE-AN. Therefore, the present exploratory study provides a starting point to investigate a potential role for proactive inhibition in SE-AN, and the extent to which proactive inhibition may be implicated across different presentations and stages of illness. Our aims were to (a) assess proactive inhibition, using a cued reaction time (RT) task, in a sample of adults with SE-AN and a healthy comparison (HC) group, and (b) explore the relationship between proactive inhibition and self-reported restrictive/avoidant eating behaviour. We predicted that those with SE-AN would demonstrate greater proactive inhibitory control compared to HCs, and that this would be positively related to intolerance of uncertainty and the severity of self-reported avoidant/restrictive eating behaviours.

## Methods

We used data collected as part of a randomised controlled feasibility trial investigating repetitive transcranial magnetic stimulation treatment for SE-AN (Trial registration: ISRCTN14329415; for details see 21, 22). The study received ethical approval from the London – City Road and Hampstead Research Ethics Committee (Reference: 15/LO/0196) and the King’s College London (KCL) Psychiatry, Nursing and Midwifery Research Ethics Subcommittee (Reference: HR-15/16-2836).

### Participants

Thirty-four community-based women (≥18 years; mean ± SD: 29.39 ± 10.31) with a current Diagnostic and Statistical Manual of Mental Disorders (DSM)-5 [1] diagnosis of AN were recruited from specialist eating disorder services in London and via online advertisements. Inclusion criteria were: aged ≥18 years; a current DSM-5 [1] diagnosis of AN; a minimum illness duration of 3 years; and completion of at least one National Institute for Health and Care Excellence [NICE; 23]-recommended specialist psychotherapy, day-patient or inpatient treatment for their eating disorder. A comparison sample of 30 healthy females (≥18 years; mean ± SD: 25.62 ± 4.08), with no current/past psychiatric illness or a family history of an eating disorder and a body mass index (BMI) within the healthy range (20–25 kg/m^2^), were recruited via online and poster advertisements at KCL. All participants were screened for eligibility (see Supplementary A for additional inclusion/exclusion criteria and screening details) and provided informed consent.

### Cued Reaction Time (RT) task

In this task (as used in [11]), a white fixation cross is presented on a screen, flanked by two empty boxes. Participants are instructed to respond as quickly as possible to the location of a visual target (large yellow dot) that appears in one of two boxes (left/right of the fixation cross) on each trial. On some trials (cued trials), the target is preceded by a spatially uninformative warning cue (the outline of the target presented in both boxes at the same time). The cue signals the upcoming presentation of the target, thereby increasing the certainty of a response being required, but gives no information as to the directionality of the response. Participants were informed that the presence of the cue was warning them that the target would soon be appearing and were reminded to only respond to the target and not the cue. A schematic of the task can be seen in Supplementary B.

This study employed a blocked design (three experimental blocks). One block is comprised only of non-cued trials (“pure” block, 40 trials). The other two blocks contain a mixture of cued and non-cued trials, with the stimulus onset asynchrony (SOA) varied on the cued trials. These mixed blocks contain 40 non-cued (0ms) trials, and 20 trials at each of the following SOAs: 100ms, 300ms and 500ms, presented in a pseudorandomised order. Participants completed the experimental blocks in a randomised, counterbalanced order.

This task assessed proactive inhibition in two ways [11, 24]. (1) “Preparation cost” reflects the extent to which one’s responses are slowed when not all stimuli in the block are targets for response (i.e., not all require a response). This is calculated by comparing the reaction times (RTs) on all trials in the pure block (i.e., “certain” go trials) to non-cued (0ms SOA) trials in the mixed block (i.e., these are “uncertain” go trials as the first visual stimulus on each trial may be either a target or a warning cue). Faster RTs in the pure block are thought to indicate that participants recognised the change in certainty of a required response and engaged in proactive inhibition during the mixed blocks. (2) “Warning benefit” is the degree to which RTs on cued trials benefit from longer SOAs (i.e., longer time to register the information provided by the warning cue: the next stimulus will be a target and therefore a response will certainly be required), calculated by comparing RTs on trials at each of the four SOAs (0ms, 100ms, 300ms, 500ms).

### Additional measures

Restrictive/avoidant eating behaviours were measured using the Restraint subscale of the Eating Disorder Examination Questionnaire [EDE-Q; 25]; the Self-Starvation Scale, a measure of compulsive food restriction [SS; 26]; and the Food Avoidance Behaviours subscale of the Fear of Food Measure [FoFM; 27]. Intolerance of uncertainty was measured using the Intolerance of Uncertainty Scale [IUS; 28]. Data from other questionnaires in the trial are reported elsewhere [22] .

### Procedure

Participants attended a research session at the Centre for Neuroimaging Sciences, Institute of Psychiatry, Psychology & Neuroscience, KCL. For the SE-AN participants, this constituted the baseline assessment of the trial, and for HCs, this was a one-off assessment. Participants’ height and weight were measured. Following an MRI scan, participants completed the cued RT task, along with additional computer tasks, on a laptop with a researcher present, and a paper questionnaire pack.

### Statistical analyses

Analyses were performed using SPSS^®^ Version 27. Demographic data were compared between groups using *t*-tests and Mann-Whitney *U* tests depending on the distribution of the data. Mean RT data on the cued RT task were positively skewed, therefore reciprocal transformations were applied and the data reversed (to maintain the original order: transformation=(1/Xi)*-1). Transformed values were entered into the following two ANOVAs. Preparation cost (i.e., the impact of learned likelihood of the next stimulus being a warning cue or target on RT) was assessed in a 2 × 2 mixed-effects ANOVA comparing RTs on non-cued trials in the mixed block (0ms SOA) with trials in the pure block (all non-cued trials) between the groups. Warning benefit (i.e., the impact of SOA on RT) was assessed in a 2 × 4 mixed-effects ANOVA comparing RTs at each of the four SOAs (0ms, 100ms, 300ms, 500ms) in the mixed blocks between the two groups. Bonferroni-corrected post-hoc *t*-tests were conducted to compare group differences in RT and accuracy on each trial type (pure, 0ms, 100ms, 300ms, 500ms). Standardised effect sizes were calculated using Cohen’s *d* and partial eta squared (η_*p*_^2^). A post-hoc sensitivity power analysis using G*power [29] indicated that the present study‘s sample size was sufficient to detect a minimum of a small effect size with a significance criterion of α = 0.05 and power = 0.80.

To explore associations between proactive inhibition indices, intolerance of uncertainty and restrictive/avoidant eating behaviours (as measured by the EDE-Q Restraint Subscale, SS, and FoFM Food Avoidance Subscale) in the SE-AN group, we performed Pearson’s or Spearman’s correlations (depending on the distribution of the data). Based on previous findings, it could be expected that intolerance of uncertainty moderates the relationship between proactive inhibition and restrictive/avoidant eating behaviours. Therefore, we also performed moderated regression analyses between each proactive inhibition index and each measure of restrictive/avoidant eating behaviours, with the continuous variable intolerance of uncertainty included as a potential moderator. For all correlational and moderation regression analyses, an index of “preparation cost” (calculated by subtracting the mean RT from non-cued trials in the mixed blocks from the pure block) and “warning benefit” (calculated by subtracting the mean RT on trials hypothesised to have the greatest benefit [i.e., longest delay between cue and target; 500ms SOA] from the trials with no benefit [0ms SOA]) were used.

## Results

Data from three participants were excluded from all analyses: one participant in each group had incomplete data on the cued RT task, and one SE-AN participant did not complete the questionnaires. Demographic characteristics and questionnaire data are shown in Supplementary File Table S1. The mean RT and accuracy percentages separately for trials in the pure block and trials at each SOA within the mixed blocks are presented in Table 1. SE-AN participants had significantly longer RTs than HCs on trials of the mixed block with 0ms (i.e., non-cued), 100ms and 300ms SOA. Accuracy was high in all conditions for both groups, with the SE-AN group showing significantly greater accuracy on the pure block than the HCs.

**Table 1:** Mean (± standard deviation) reaction times (ms) and accuracy (%) for each trial category for the severe and enduring anorexia nervosa and healthy comparison participants

	Mean ± SD (95% CI) Reaction Time (ms)				Mean ± SD (95% CI) Accuracy (%)		
	SE-AN	HC	Group comparison		SE-AN	HC	Group comparison
Pure	389.24 ± 52.96 (370.46-408.02)	372.24 ± 39.90 (357.06-387.41)	*t*(60)=-1.41, *p* = 0.163, *d* = 0.36		99.55 ± 0.98 (99.20-99.89)	98.19 ± 2.66 (97.18–99.20)	*t*(60)=-2.72, *p* < 0.010, *d* = 0.68
0ms	452.74 ± 59.40 (431.67–473.80)	417.33 ± 44.08 (400.57–434.10)	*t*(60)=-2.63, *p* < 0.010, *d* = 0.68		99.02 ± 2.16 (98.25–99.78)	98.97 ± 1.83 (98.27–99.66)	*t*(60)=-0.10, *p* = 0.923, *d* = 0.02
100ms	419.21 ± 55.31 (399.60-438.82)	379.64 ± 30.06 (368.30-391.07)	*t*(60)=-3.43, *p* < 0.001, *d* = 0.89		97.27 ± 6.14 (95.10-99.45)	98.10 ± 2.81 (97.04–99.17)	*t*(60)=-0.67, *p* = 0.506, *d*=-0.17
300ms	436.94 ± 61.18 (415.25-458.64)	397.70 ± 42.90 (381.38-414.02)	*t*(60)=-2.89, *p* < 0.005, *d* = 0.74		97.12 ± 4.15 (95.65–98.59)	96.72 ± 6.98 (94.07–99.38)	*t*(60)=-0.28, *p* = 0.783, *d* = 0.07
500ms	379.41 ± 61.96 (357.44-401.38)	353.02 ± 48.89 (357.44-401.38)	*t*(60)=-1.84, *p* = 0.070, *d* = 0.47		98.48 ± 2.33 (97.68–99.31)	98.45 ± 2.71 (97.42–99.48)	*t*(60)=-0.57, *p* = 0.955, *d* = 0.01

Abbreviations: SE-AN = severe and enduring anorexia nervosa; HC = healthy comparison; SD = standard deviation; 95% CI = 95% Confidence interval for mean

### Preparation cost

Comparing RTs on trials in the pure block with non-cued trials in the mixed block revealed a main effect of Block (*F*(1, 60) = 187.896, *p* < 0.001, η_*p*_^2^ = 0.758), such that participants were slower to respond to non-cued trials in the mixed block compared to the pure block, and a main effect of Group (*F*(1, 60) = 4.082, *p* = 0.048, partial η_*p*_^2^ = 0.064), whereby SE-AN participants had slower RTs overall compared to HCs. No significant Group*Block interaction was observed (*F*(1, 60) = 2.237, *p* = 0.140, partial η_*p*_^2^ = 0.036). However, as can be seen in Table 1, group differences in RTs reached statistical significance in the mixed block (*t*(60)=-2.63, *p* < 0.010, *d* = 0.68), but not the pure block (*t*(60)=-1.41, *p* = 0.163, *d* = 0.36).

### Warning benefit

A 2 × 4 Group*SOA ANOVA on trials in the mixed blocks only revealed a significant main effect of SOA, with participants responding fastest on trials with the longest (500ms) cue-target SOA, and slowest in the non-cued (0ms) trials (*F*(1.485, 89.111) = 106.007, *p* < 0.001, η_*p*_^2^ = 0.639; see Supplementary D for post-hoc comparisons). A main effect of Group was also observed (*F*(1, 60) = 7.322, *p* = 0.009, η_*p*_^2^ = 0.109), with the SE-AN group demonstrating significantly slower RTs at each SOA compared to the HCs (Table 1). There was no significant Group*SOA interaction (*F*(1.485, 89.111) = 0.378, *p* = 0.624, η_*p*_^2^ = 0.006).

### Relationship between proactive inhibition, intolerance of uncertainty, and restrictive/avoidant eating behaviours

In the SE-AN sample, no measures of restrictive/avoidant eating behaviours nor intolerance of uncertainty were related to warning benefit or preparation cost (*p* > 0.05; see Supplementary File Table S2). Intolerance of uncertainty was significantly and positively associated with scores on the EDE-Q Restraint subscale and the SS (*p* < 0.05). Moderation regression analyses did not provide evidence to suggest that the degree of association between proactive inhibition and restrictive/avoidant eating behaviours was dependent upon individual differences in intolerance of uncertainty (*p* > 0.05).

## Discussion

Proactive inhibition (i.e., a pre-emptive withholding or slowing of responses under uncertainty) may represent a neurocognitive factor involved in AN. This study compared proactive inhibition between people with SE-AN and HCs and explored whether proactive inhibition was associated with self-reported restrictive/avoidant eating behaviours. We predicted that individuals with SE-AN would show greater proactive inhibition than HCs, and that this would be positively related to restrictive/avoidant eating behaviours and to intolerance of uncertainty. Both groups demonstrated proactive inhibition, i.e., both responded more slowly on non-cued trials in the mixed blocks compared to the pure block (preparation cost), and generally responded more quickly as the delay between the cue and target onset increased (warning benefit). Although a main effect of group was observed, with the SE-AN group having slower response times than HCs, no significant interaction was found, suggesting that those with SE-AN did not show differences in the *extent* to which they exercised proactive inhibition during the task. We found no association between proactive inhibition, intolerance of uncertainty, or restrictive/avoidant eating behaviours.

Consistent with our previous study in AN [11], the data revealed that across all trial types, the SE-AN group generally responded more slowly than HCs. This suggests a general slowing of motor/behavioural responses in the SE-AN group. However, an ANOVA did not identify a significant interaction term, perhaps due to the ANOVAs not including a comparison across all 5 trial types. Post-hoc comparisons for each trial type only identified significant group differences under conditions of uncertainty (at 0ms, 100ms and 300ms SOA in the mixed block) and not in the absence of uncertainty (500ms SOA or pure block). This is similar to our previous findings [11], in which post-hoc comparisons at each SOA revealed slower responses in AN compared to HC participants across all SOAs in the mixed block but not the pure block. The present post-hoc findings are not consistent with the notion of a general slowing of behaviour and may instead suggest that individuals with SE-AN may exercise greater proactive inhibition (i.e., more pre-emptive slowing) in the context of uncertainty relative to HCs. Research using alternative measures of proactive inhibition would help characterise this further. If true, it may be that people with SE-AN tend towards a more cautious approach in their responding, perhaps prioritising accuracy over speed. Individuals with AN display perfectionistic tendencies [30] and attribute greater importance to accuracy in their decision-making process [31], e.g., spending more time checking their work compared to healthy individuals [32]. Also, network analyses have highlighted ‘concern over mistakes’ as a central symptom in AN [33]. However, we did not formally assess perfectionism in our study.

We explored the relationship between proactive inhibition, intolerance of uncertainty and restrictive/avoidant eating behaviours. We previously found that group differences in proactive inhibition emerged after covarying for intolerance of uncertainty [11], but this was not seen in the present study. This may be due to sample differences as our previous study involved four participant groups (HC, AN, BN and BED). Moreover, the AN group in our previous study was more heterogeneous in terms of illness chronicity: differences in findings between our studies may relate to differences in the way proactive inhibition might be involved across stages of illness severity. Studies comparing proactive inhibition across groups at different stages of illness would help to address this.

We found that greater intolerance of uncertainty was associated with more severe symptoms of restrictive eating, i.e., consistent with previous research [18–20]. However, proactive inhibition was not related to intolerance of uncertainty nor to measures of restrictive/avoidant eating behaviours (in both correlation analyses and moderation regressions with intolerance of uncertainty included as a moderator). This suggests that proactive inhibition is unlikely to be a specific contributing mechanism to restrictive/avoidant eating behaviours in SE-AN and that individual differences in intolerance of uncertainty are not responsible for this lack of association. Whilst a link between contextual uncertainty and the expression of proactive inhibition has been established [34], it is likely that the task we used does not tap into the same processes that are assessed with intolerance of uncertainty questionnaires (e.g., sensitivity to delayed or uncertain outcomes). Efforts to characterise proactive inhibition in AN may benefit from inclusion of explicit feedback (i.e., outcome and running score), induction of a goal conflict (i.e., between accurate performance and the speed with which stimuli are removed from the screen), or disorder-relevant stimuli. Such experimental manipulations are likely to more closely tap into relevant behavioural traits of people with AN [35].

This study has explored proactive inhibitory control in individuals with SE-AN. However, we did not include a comparison group with a less severe and enduring form of AN. Therefore, it is unclear if our findings are specific to SE-AN, or a marker of AN more broadly. Studies using longitudinal designs and between-group comparisons of individuals with SE-AN to those with less severe and/or more recent onset AN will be important in evaluating the specificity and influence of proactive inhibition across illness stages. Such studies will help to characterise if and how proactive inhibition changes as illness progresses, its association with symptom maintenance or symptom change, and whether a particular profile of proactive inhibition is a predisposing phenotype for a more enduring form of or future illness.

The study has some limitations. The SE-AN group included individuals with both subtypes of AN (AN restricting [AN-R *n* = 21]; AN binge-eating/purging [AN-BP *n* = 12]). Binge eating and purging behaviours have been associated with impulsivity and inhibitory control difficulties [36], whereas those with AN-R are proposed to have superior inhibitory control [8]. Therefore, combining AN subtypes with opposing levels of response inhibition may have influenced the pattern of proactive inhibition and its relationship with restrictive eating. In addition, we used a simple, neutral stimulus in a non-disorder-specific context and it may be that differences in proactive inhibition emerge in more anxiety-provoking contexts. For example, greater inhibitory control deficits in the context of disorder-relevant, compared to general, stimuli have been reported in BN [36]. Thus, studies should explore whether response inhibition is manifested differently in the context of food compared to neutral or non-disorder-specific stimuli in AN [e.g., 24, 37]. It may be that differences in proactive inhibition are more evident at a neural rather than behavioural level. Indeed, several neuroimaging studies in AN have found differences in inhibition-related neural activity in the absence of behavioural differences [12, 38, 39]. Research is needed into whether differences in the neural correlates of proactive inhibition are similarly observed in individuals with SE-AN.

## Conclusions

We explored proactive inhibition and its relationship to restrictive/avoidant eating behaviour and to intolerance of uncertainty in individuals with SE-AN. Although not evident in our ANOVA, post-hoc inspection of the data revealed that compared to HC, individuals with SE-AN show greater slowing of responses specifically under conditions of uncertainty. In the SE-AN group, however, proactive inhibition does not appear to be related to self-reported restrictive/avoidant eating behaviours or to intolerance of uncertainty. We propose that proactive inhibition may be more involved in decision making and that the slowing of responses may reflect a prioritisation of accuracy. This could be related to perfectionism, and therefore, proactive inhibition may be a more relevant target when addressing rigidity and perfectionism rather than eating behaviours. Research using longitudinal studies, and/or between-group comparisons of individuals with AN at different illness stages will be helpful in determining the way in which proactive inhibition is involved in the development and maintenance of SE-AN.

## Electronic supplementary material

Below is the link to the electronic supplementary material.

Additional File 1: Supplementary A – Eligibility criteria and screening; Supplementary B – Schematic diagram of the cued reaction time task; Supplementary C – Participant demographics and clinical characteristics; Supplementary D – Post-hoc *t*-tests exploring the main effects of stimulus onset asynchrony (SOA) and group on warning benefit; Supplementary E – Correlations between proactive inhibition, restrictive/avoidant eating behaviours and intolerance of uncertainty

## Data Availability

The datasets generated and/or analysed during the current study are not publicly available but are available from the corresponding author on reasonable request.

## Abbreviations

AN: Anorexia nervosa
BMI: Body mass index
DSM: Diagnostic and Statistical Manual of Mental Disorders
ED: Eating disorder
EDE-Q: Eating Disorder Examination Questionnaire
FoFM: Fear of Food Measure
HC: Healthy comparison
IUS: Intolerance of Uncertainty Scale
KCL: King’s College London
MRI: Magnetic resonance imaging
RT: Reaction time
SE-AN: Severe and enduring anorexia nervosa
SOA: Stimulus onset asynchrony
SS: Self-Starvation Scale
