# Supplementary material for: Exploring the relationship between proactive inhibition and restrictive eating behaviours in severe and enduring anorexia nervosa (SE-AN)
Source: J Eat Disord. 2025 Jan 3;13:1. doi: 10.1186/s40337-024-01165-y (PMC11699635; doi:10.1186/s40337-024-01165-y)
Supplement: Supplementary file 1 — Additional File 1: Supplementary A – Eligibility criteria and screening; Supplementary B – Schematic diagram of the cued reaction time task; Supplementary C – Participant demographics and clinical characteristics; Supplementary D – Post-hoc t-tests exploring the main effects of stimulus onset asynchrony (SOA) and group on warning benefit; Supplementary E – Correlations between proactive inhibition, restrictive/avoidant eating behaviours and intolerance of uncertainty [file 40337_2024_1165_MOESM1_ESM.docx]

# Supplementary A – Eligibility criteria and screening

Additional eligibility criteria for the severe and enduring anorexia nervosa (SE-AN) group were: (a) a minimum illness duration of 3 years; (b) completion of at least one National Institute for Health and Care Excellence (NICE; 2017)-recommended specialist psychotherapy, day-patient or inpatient treatment for their eating disorder; and (c) no repetitive transcranial magnetic stimulation (rTMS) contraindications. For all participants, additional exclusion criteria were magnetic resonance imaging contraindications.

Participants were screened for eligibility via a telephone assessment: group membership (healthy comparisons [HC]/SE-AN) was confirmed using the Eating Disorder Diagnostic Scale (EDDS; Stice et al., 2000), and the absence of any psychiatric disorders in HCs was assessed using the EDDS and the researcher version of the Structured Clinical Interview for DSM-IV Axis I Disorders Screening Module (SCID-IV; First et al., 2002).

# Supplementary B – Schematic diagram of the cued reaction time task


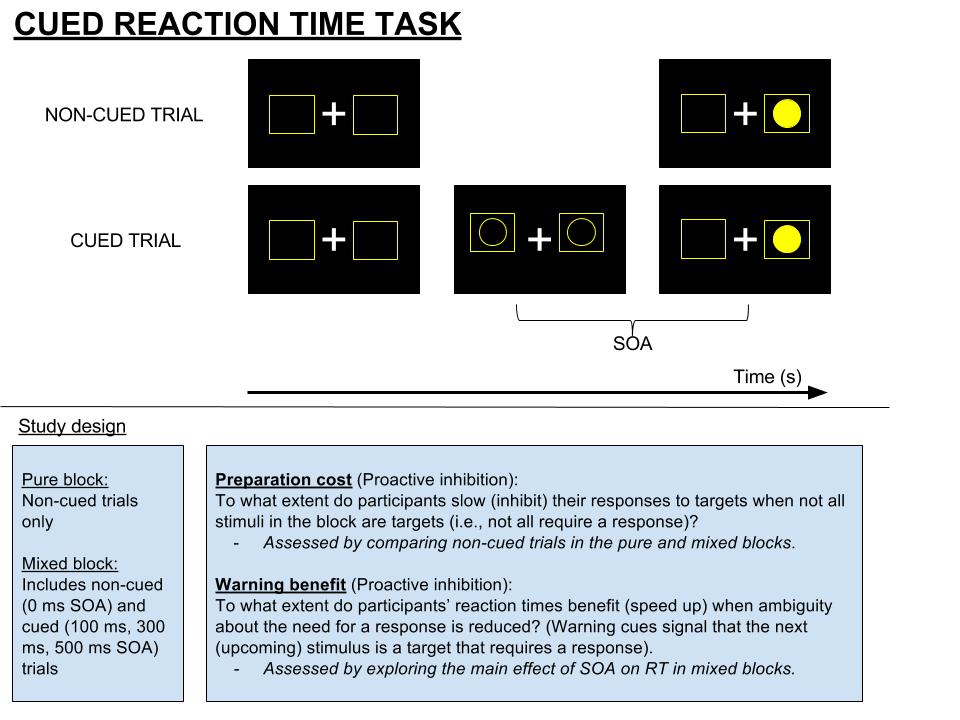


Note: SOA = stimulus onset asynchrony; RT = reaction time; s = seconds; ms = milliseconds

# Supplementary C – Participant demographics and clinical characteristics

**Table S1.** Demographics and questionnaire data on anxiety, intolerance of uncertainty and avoidant/restrictive eating behaviours for the severe and enduring anorexia nervosa and healthy comparison participants.

|  |  | **SE-AN (n=33)**  **(mean ± SD)** | **HC (n=29)**  **(mean ± SD)** | **Group comparison** |
| --- | --- | --- | --- | --- |
| **Age (years)** | | 29.39 ± 10.31 | 25.62 ± 4.08 | *U*=556.5, *z*=1.103, *p*=0.270 |
| **BMI (kg/m^2^)** | | 16.09 ± 1.45 | 21.92 ± 1.56 | *t*(60)=15.266, *p*<0.001 |
| **Illness duration (years)** | | 13.80 ± 10.80 | - | - |
| **IUS^#^** | Total | 92.00 ± 22.30 | 45.97 ± 11.32 | *U*=898.5, *z*=6.277, *p*<0.001 |
| **EDEQ^#^** | *Restraint* | 3.96 ± 1.56 | 0.39 ± 0.60 | *U*=908.5, *z*=6.473, *p*<0.001 |
| **SS^#^** | Total | 54.28 ± 23.40 | 0.66 ± 1.17 | *U*=928, *z*=6.824, *p*<0.001 |
| **FoFM^#^** | *Food Avoidance Behaviours* | 29.69 ± 7.39 | 6.62 ± 1.02 | *U*=927, *z*=6.795, *p*<0.001 |

**^#^**Data missing from one AN participant. Abbreviations: SE-AN = severe and enduring anorexia nervosa; HC = healthy comparison; SD = standard deviation; BMI = body mass index; IUS = Intolerance of Uncertainty Scale; EDE-Q = Eating Disorder Examination Questionnaire; SS = Self-Starvation Scale; FoFM = Fear of Food Measure.

# Supplementary D – Post-hoc t-tests exploring the main effects of stimulus onset asynchrony (SOA) on warning benefit

Across the whole sample, participants responded more quickly as stimulus onset asynchrony (SOA) increased (**Table S2**). The main effect of SOA was further explored using post-hoc paired samples *t*-tests, which were corrected for multiple comparisons using Bonferroni correction. Participants responded fastest on the trials in which the target appeared after 500ms SOA compared to all other SOAs (0ms SOA: *t*(61)=12.880, *p*<0.001, *d*=1.64; 100ms SOA: *t*(61)=8.739, *p*<0.001, *d*=1.11; 300ms SOA: *t*(61)=12.716, *p*<0.001, *d*=1.89). Participants were slowest to respond on non-cued trials (0ms vs. 100ms SOA: *t*(61)=10.63047, *p*<0.001, *d*=1.35; 0ms vs. 300ms SOA: *t*(61)=4.948, *p*<0.001, *d*=0.63). In line with our previous reports, participants performed faster on trials with 100ms SOA compared to 300ms SOA (*t*(61)=-6.653, *p*<0.001, *d=*-0.84).

**Table S2. Mean (± standard deviation) reaction times (ms) for each trial category across the whole sample.**

| **Trial category (SOA)** | **Mean (± SD) reaction time (ms)** |
| --- | --- |
| 0ms | 436.18 (55.32) |
| 100ms | 400.70 (49.15) |
| 300ms | 418.59 (56.55) |
| 500ms | 367.07 (57.33) |

Abbreviations: SOA = stimulus onset asynchrony; SD = standard deviation

# Supplementary E – Correlations between proactive inhibition, restrictive/avoidant eating behaviours and intolerance of uncertainty

**Table S2.** Correlations assessing the relationship between restrictive eating behaviours, intolerance of uncertainty, and proactive inhibition in the severe and enduring anorexia nervosa group only.

|  |  | 1 | 2 | 3 | 4 | 5 |
| --- | --- | --- | --- | --- | --- | --- |
| 1 | IUS Total | - | - | - | - | - |
| 2 | EDE-Q Restraint | ***r*=0.370, *p*=0.037** | *-* | - | - | - |
| 3 | Self-Starvation | ***r*=0.422, *p*=0.016** | ***r*=0.710, *p*<0.001** | - | - | - |
| 4 | FoFM Food Avoidance | *r_s_*=0.266, *p*=0.142 | ***r_s_*=0.485, *p*=0.005** | ***r_s_*=0.436, *p*=0.013** | - | - |
| 5 | Warning Benefit | *r*=0.264, *p*=0.145 | *r*=-0.108, *p*=0.556 | *r*=0.111, *p*=0.546 | *r*=0.090, *p*=0.623 | - |
| 6 | Preparation Cost | *r*=0.157, *p*=0.390 | *r*=-0.054, *p*=0.768 | *r*=0.095, *p*=0.603 | *r*=0.115, *p*=0.529 | ***r*=0.733, *p*<0.001** |

Bold font indicates statistical significance at *p* < 0.05. Abbreviations: DASS-21 = Depression Anxiety and Stress Scales – Version 21; IUS = Intolerance of Uncertainty Scale; EDE-Q = Eating Disorder Examination Questionnaire; FoFM = Fear of Food Measure
